# The Role of Lipids in Allosteric Modulation of Dopamine D_2_ Receptor—In Silico Study

**DOI:** 10.3390/molecules27041335

**Published:** 2022-02-16

**Authors:** Justyna Żuk, Damian Bartuzi, Przemysław Miszta, Agnieszka A. Kaczor

**Affiliations:** 1 Department of Synthesis and Chemical Technology of Pharmaceutical Substances with Computer Modelling Laboratory, Faculty of Pharmacy, Medical University of Lublin, 4A Chodźki St., PL-20093 Lublin, Poland; j.siudem@gmail.com (J.Ż.); damian.bartuzi@umlub.pl (D.B.); 2 Faculty of Chemistry, Biological, Chemical Research Centre, University of Warsaw, PL-02093 Warsaw, Poland; pmfenix@wp.pl; 3 School of Pharmacy, University of Eastern Finland, Yliopistonranta 1, P.O. Box 1627, FI-70211 Kuopio, Finland

**Keywords:** allosteric modulators, dopamine D_2L_ receptor, GPCRs, lipid rafts, molecular dynamics

## Abstract

The dopamine D_2_ receptor, belonging to the class A G protein-coupled receptors (GPCRs), is an important drug target for several diseases, including schizophrenia and Parkinson’s disease. The D_2_ receptor can be activated by the natural neurotransmitter dopamine or by synthetic ligands, which in both cases leads to the receptor coupling with a G protein. In addition to receptor modulation by orthosteric or allosteric ligands, it has been shown that lipids may affect the behaviour of membrane proteins. We constructed a model of a D_2_ receptor with a long intracellular loop (ICL3) coupled with G_iα1_ or G_iα2_ proteins, embedded in a complex asymmetric membrane, and simulated it in complex with positive, negative or neutral allosteric ligands. In this study, we focused on the influence of ligand binding and G protein coupling on the membrane–receptor interactions. We show that there is a noticeable interplay between the cell membrane, G proteins, D_2_ receptor and its modulators.

## 1. Introduction

Lipid membranes are not only the physical boundaries separating cells from the external environment [1], but they constitute fully specialized lipid–protein structures that perform many different functions in living organisms [2]. They enable the selective transport of many essential substances and drugs to the cells. In the classical fluid mosaic model, biological membranes were assumed to be homogeneous mixtures of lipids and proteins, with their components moving freely within each layer [3]. However, studies based on the conformational order of lipids and differences in their short-range translation proved the existence of heterogeneity and structurally and functionally organized regions in the biological membrane, which are termed lipid rafts [4]. They are enriched in sphingolipids with a predominance of simple hydrocarbon chains, cholesterol, glycosphingolipids, and proteins forming specific platforms or lipid microdomains [5]. It was found that different ratios of lipids in the rafts affect the fluidity of the membrane [6,7,8,9,10,11].

Lipid rafts are involved in many important cellular processes. Their role in protein sorting and membrane transport was confirmed in a number of studies [9,12]. Furthermore, they contain different types of proteins that participate in signal transduction. According to Simons and Toomre [13], lipid rafts are specific platforms for given receptors where activation takes place through ligand binding. The concept of rafts to serve as signal transduction platforms stems not only from their enrichment with signalling molecules but also from the observation that some receptors must be transferred to the lipid rafts after ligand binding to initiate a cellular response [13].

G protein-coupled receptors (GPCRs) are transmembrane proteins that induce relatively rapid and highly specific responses to stimuli. Available data suggest that GPCR signalling components are organized in raft microdomains. Lipid rafts seem to regulate GPCR signal transduction in eukaryotic cells [14]. GPCRs are a group of important drug targets, and it has been proposed that the lipid membrane can facilitate the binding of medicinal compounds to the target proteins [15]. These ligand–receptor interactions may lead to alterations in membrane thickness, lipid spontaneous curvature and dynamics, lipid packing density or membrane structure. All these properties will, in turn, influence the structure and function of membrane proteins and, ultimately, their biological function [16,17].

**Figure 1:**
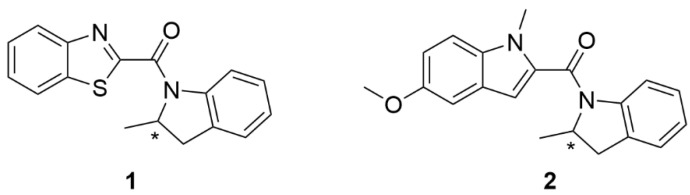
Structural formulas of the studied compounds **1** and **2.** The asterisk (*) denotes a chiral carbon.

In our previous work [18] we used molecular docking methods to identify the part of the dopamine D_2L_ receptor (belonging to GPCRs) that is most probably involved in the binding of allosteric ligands **1** [19] and **2** [18], see Figure 1. Both enantiomers of compound **1** (**R1** and **S1**) and compound **2** (**R2** and **S2**) were studied. **R1** is a positive allosteric modulator of the D_2L_ receptor, while **S1** is its negative allosteric modulator [18,19]. **R2** and **S2** were proposed to be silent allosteric ligands [18]. The position of the allosteric pocket was dependent on the type of model used. In systems of dopamine D_2_ receptor in complex with G_i__α__1_ (DG1), all ligands bound deeper inside the receptor, just above dopamine, while ligands in systems of dopamine D_2_ receptor in complex with G_i__α__2_ (DG2) bound closer to the extracellular part of the receptor. During MD simulations, allosteric modulators bound to DG2 migrated into lower binding pockets. As a reference, systems without the modulator (dopamine-bound receptor) were also simulated. We examined RMSD values for individual helices which allowed to define the most dynamic receptor structures. In particular, TM5, TM6 and TM7 movements proved important in the study of modulation of the allosteric ligands used. The studies have shown that in simulations with the **R1** modulator, significant bending and rotating of TM6 towards the cytoplasmic side, which keeps the receptor in the active state, were observed. Because of rotameric transitions of Y5.58 and Y7.53 (Ballesteros−Weinstein notation [20]) their side chains can be placed within the space emptied by the outward movement of TM6. Rearrangement of these residues appears to stabilize the receptor in its active state by a structural water-mediated hydrogen bond network. In contrast, modulator **S1** caused a larger fluctuation and increase the distance between Y5.58 and Y7.53. Furthermore, in the case of **S1**, the spatial organization of TM5, TM6 and TM7 differs significantly. Regarding all analyses with compound **2**, conformations of complexes assume intermediate conformations, in between extremes explored by **R1** and **S1**, which, together with in vitro results [18], supports the conclusion that compound **2** does not affect the conformational states of the protein after binding and plays a role of a neutral allosteric ligand.

In the present study, we focus on the changes in membrane properties and provide a detailed description of how compounds **R1**, **S1, R2** and **S2** bound to D_2L_ dopamine receptor in complex with G_i__α__1_ or G_i__α__2_ proteins (R1G1, R1G2, S1G1, S1G2, R2G1, R2G2, S2G1 and S2G2) affect the surrounding membrane. The bilayer composition includes nine types of lipids in the proportions appropriate for membrane rafts, containing cholesterol, sphingomyelin, DOPE, DOPC, DOPS, PLPC, POPC, POPE and POPG. 

## 2. Results

The area per lipid (APL) is an important parameter that provides structural insights into lipid bilayer perturbation. In this study, we aimed to check the effect of modulator binding on the ligand–receptor–membrane systems using **R1**, **S1**, **R2** and **S2** compounds. Both the total APL and the APL of each lipid species separately were calculated (Table 1, Figures S1 and S2). The simulations show that the values for the total APL are the highest in the presence of **R1**. Exact values are as high as 58.46 ± 0.20 Å^2^ for the G_i1_ complex and 56.89 ± 0.27 for the G_i2_ complex. Comparing simulations for modulator **S1** and simulations in the absence of modulators, the dopamine–receptor complexes show a relatively large APL with a value of 50.00 Å^2^, while values for modulator **S1** are 48.55 ± 0.33 Å^2^ for the G_i1_ complex and 48.40 ± 0.20 Å^2^ for the G_i2_ complex which indicates that these systems are relatively less perturbed compared to the complexes with modulator **R1**. It is worth noting that the values of average APL for DOPE, DOPC, DOPS, PLPC, POPC, POPE and POPG are higher for modulator **R1** than modulators **S1**, **R2** and **S2**. In the absence of modulators, the values are usually higher than those observed in the **S1**-bound complex and close values seen in complexes containing **R2** and **S2**. Notably, in the case of systems with G_i1_, the fluctuations are higher than in the systems with G_i2_ (Table 1). The standard deviations of APL values (Table 2) show that higher fluctuations occur in the presence of modulators as well as in systems with the G_i1_ protein.

The higher total area per lipid values in **R1** simulations are interesting, given that most area per lipid values for particular lipid types are similar across all simulations. Notably, only DOPE and POPG APL values are altered in **R1** simulations. Importantly, the MEMBPLUGIN code in its present version does not account for the area occupied by the receptor, which should be considered when analysing these results. However, while excluding protein from APL calculations may affect APL values, it is not likely to result in alterations in the ratios of APLs of different lipid species between membranes, as seen for DOPE and POPG.

**Figure 2:**
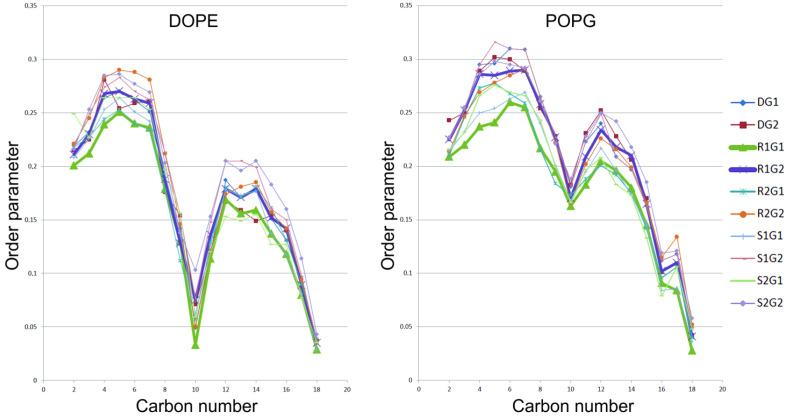
SCD order parameter values measured for DOPE and POPG in all simulations. R1 complexes were shown as bold green and blue lines.

Altered behaviour of DOPE and POPG is also reflected in S_CD_ order parameter values, which is presented in Figure 2. S_CD_ is a measure of the mobility, and orientation of the C-H bonds is the lipid order parameter [21]. This parameter quantifies the order of lipids’ hydrocarbon tails by averaging angle values per each C-H bond with respect to the z-axis in each lipid acyl tail over a given lipid moiety in the bilayer [22]. 

Table 3 shows the lateral diffusion coefficient along the bilayer plane, perpendicular to the z-axis of cholesterol and DOPC molecules in each system. Diffusion coefficients (D_2D_) of lipids were estimated by calculation of the mean square displacements (MSD) of the lipids using the Einstein relationship, which states that the average squared deviation of a particle’s position is proportional to its displacement time [23]. The calculated diffusion coefficient was obtained by the slope of the curves presented in Figure S3, where the MSD of lipid moieties was presented over time.

With regards to the **S1** modulator, the cholesterol diffusion coefficient is the smallest in comparison with the ones in the other systems, indicating that in this system, cholesterol does not diffuse so freely along the membrane plane. As for the value of the DOPC diffusion coefficient, it depends on the type of G protein. The values for individual modulators are lower for the G_i1_ protein and higher for G_i2_. The largest discrepancy between the values for individual G protein subtypes appears in the complex with **S1** and **S2** modulators. 

**Figure 3:**
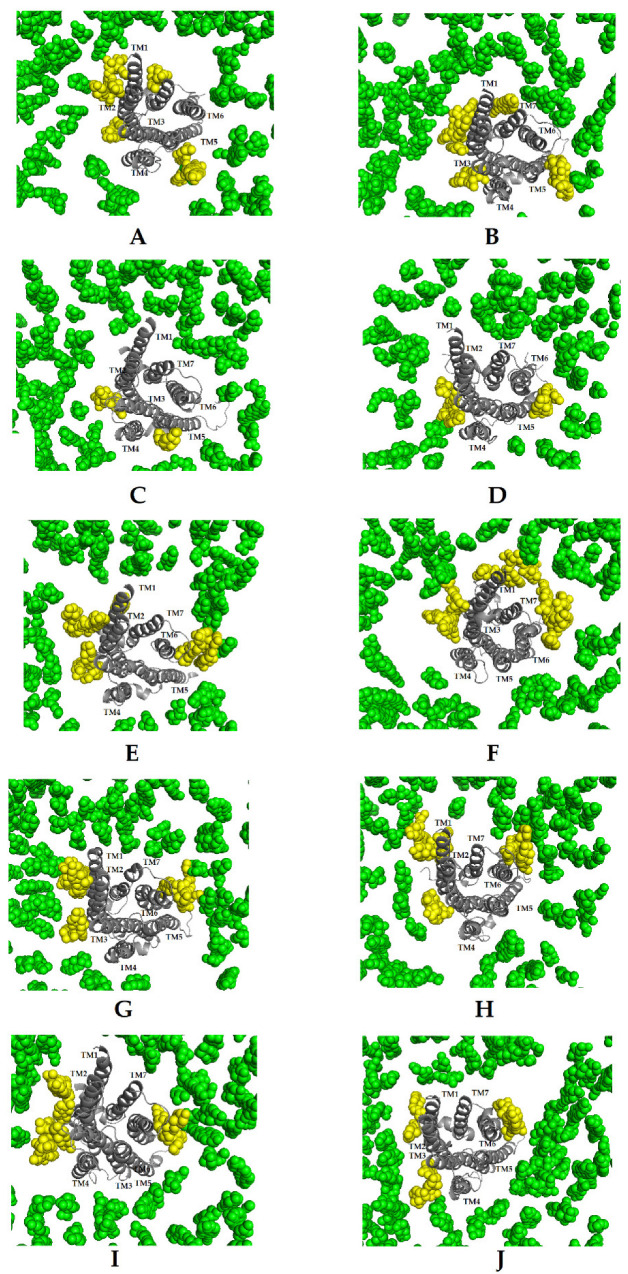
Cholesterol occupancy sites at the TM helices (showed as a grey ribbon) of the R1G1 (**A**), R1G2 (**B**), S1G1 (**C**), S1G2 (**D**), R2G1 (**E**), R2G2 (**F**), S2G1 (**G**), S2G2 (**H**), DG1 (**I**) and DG2 systems (**J**). Atoms of cholesterol molecules are shown as green spheres. The high-density cholesterol sites nearest receptor are showed as a yellow clusters.

The binding site of the cholesterol in the receptor consists of four key amino acid residues and is defined as a CCM motif [24,25,26,27] (Y2.41, K4.39, I4.46 and W4.50 in D_2_ dopamine receptor). In all the systems, two cholesterol molecules occupy roughly the same position across two transmembrane helices—TM2 and TM4. Figure 3 shows the last snapshot of MD simulations of all systems with the distribution of the cholesterol molecules oriented around the dopamine D_2_ receptor. In the case of **R1,** four main clusters can be observed in the regions of TM1–TM2, TM2–TM4, TM5 and TM7 (Figure 3A,B). In simulations with **S1,** there are two main clusters in the regions of TM2–TM4 and TM5 (Figure 3C,D). Meanwhile, in simulations with **R2, S2,** DG1 and DG2, there are three clusters located near TM1, TM2–TM4 and TM6 (Figure 3E–H). 

**Figure 4:**
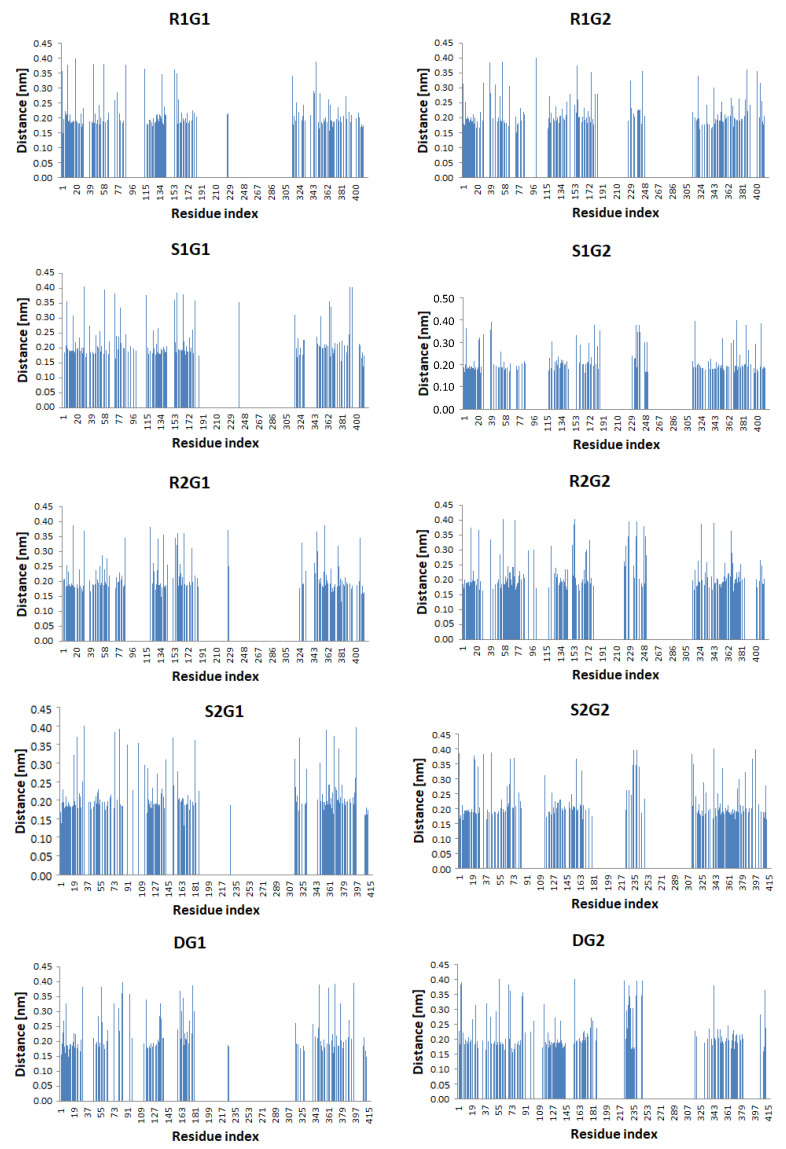
Probability of intermolecular contacts between the cholesterol and the dopamine D_2_ receptor. The results were investigated throughout the 1 µs MD simulation.

Intermolecular cholesterol—D_2_ receptor interactions were also analysed through the minimum distance measurement for any pair of atoms. Figure S4 shows the time evolution of the number of contacts of cholesterol and protein within a given distance of 0.6 nm. These plots and the average minimum distance between any pair of atoms, shown in Table 4, indicate that the process of cholesterol adsorption is slightly different for simulated systems. In R1G1 and R1G2 systems, the average number of close contacts between the receptor and cholesterol is the highest among all simulations (11,645 and 16,859, respectively), with the lowest average of minimum distance. The average number of close contacts in systems without modulators is noticeably lower (4953 and 6597, respectively). The probabilities of close contact are shown in Figure 4, where contact is registered when the distance becomes smaller than 0.40 nm. There are three main groups of contacts noticeable on each diagram. The first group, with residues numbered 1–77, corresponds to TM1, TM2, and the top of TM3. The second group (residues: 115–180) consists of TM5 and TM6 fragments, including a small part of ICL3. The third group (316–410) are the amino acids of TM7 and helix 8. Interestingly, only in simulations with the G_i2_ protein, the fourth group appears in the range of residue numbers 220–250. These residues correspond to the ICL3 loop, and their distances to cholesterol atoms are >0.4 nm. The number of residues in close contact with cholesterol was also calculated (Table 4). Notably, the highest number of residues in close contact with cholesterol is found in the R1G1 and R1G2 systems (169 and 176, respectively). On the other hand, the lowest values are found in the DG1 and DG2 systems (123 and 134, respectively). Among the modulator-bound complexes, the lowest values are seen in negatively modulated proteins (S1G1—149 and S1G2—151).

**Figure 5:**
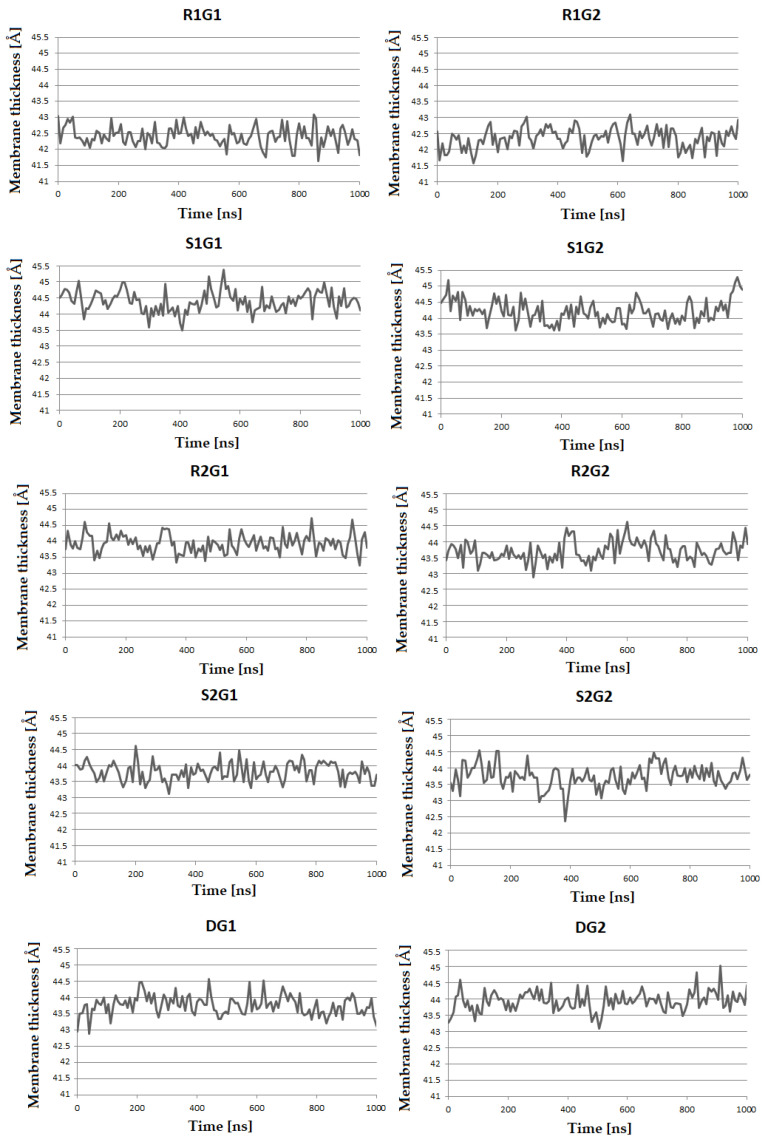
Average membrane thickness, computed for all bilayer systems.

The calculated membrane thickness values vary depending on the modulator type in the studied model systems. The plots in Figure 5 show fluctuations in membrane thickness during 1µs MD simulations. A significant difference is observed in simulations with modulators **R1** and **S1**. The estimated average membrane thickness for R1G1 and R1G2 (42.42 Å and 42.35 Å respectively) was found to be the lowest among all simulations, while simulations of the membrane thickness for S1G1 and S1G2 (44.40 Å and 44.22 Å respectively) show the highest values. In the other systems with **R2** and **S2** modulators, average membrane thickness was found to be 43.93 Å for R2G1, 43.70 Å for R2G2 Å, 43.80 Å for S2G1 and 43.74 Å for S2G2, which indicates no significant difference between these models and models in the absence of any modulator (43.76 Å for DG1 and 43.93 Å for DG2). 

**Figure 6:**
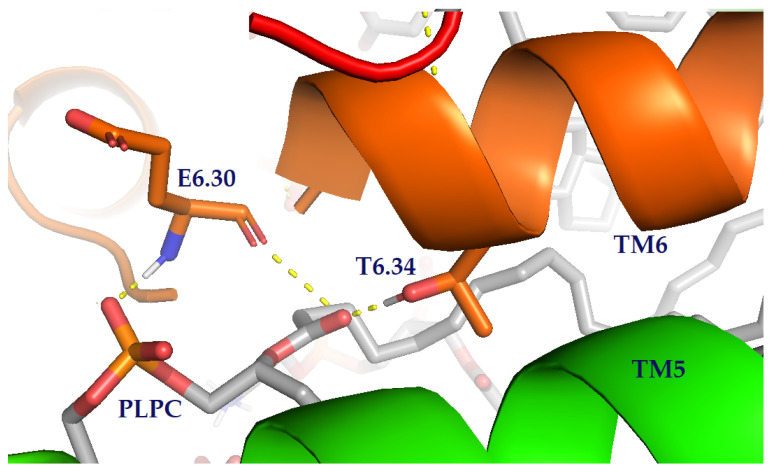
Crucial lipid–protein interactions of the dopamine D_2_ receptor with the key residues and lipids highlighted as sticks. The structures of helices are shown as ribbons. Hydrogen bonds are marked as yellow dashes.

To get further understanding of the membrane behaviour, the protein–lipid interactions in the final frames of simulations were measured. We studied the interaction of lipids with the 7TM receptor. We found in our previous study that TM6 movement and rotation depend on the type of allosteric modulation [18], which is consistent with literature reports [28]. The interactions of the cytoplasmic part of TM6 turned out to be the most significant. Only in simulations with the **R1** was an interaction of T6.34 and E6.30 with the membrane lipids found (Figure 6). This may be due to the specific bending of this helix in the presence of the modulator. In all simulations, we observed interactions of R1.59, R4.41, R5.66 and K6.32 with the inner leaflet of the bilayer.

## 3. Discussion

The interactions between proteins and lipid membranes in the presence of allosteric modulators are important for understanding of the dynamicity and function of the GPCRs [29,30,31]. Elucidating the mechanism responsible for the protein–lipid interactions or conformational changes in the lipid bilayer may lead to improvements in the field of drug design. In our research, we studied what changes occur in the lipid membrane as a result of conformational changes of the dopamine D_2L_ receptor in complex with a negative, positive or neutral allosteric ligand. The recent cryo-EM structure of dopamine D_2_R in its active state (PDB ID: 6VMS [32]) provides quite detailed information on the structure of the receptor and its activation mechanisms. However, this work is based on the modelling of the full D_2L_ receptor, including the ICL3 loop, and 1 μs MD simulations. In order to compare the experimental structure with our model, we calculated the RMSD for the 8-helical Cα atoms: 1.74 Å for DG1 and 2.29 Å for DG2 as previously reported [18]. 

In order to characterize the changes of the properties of the lipid environment, we calculated the area per lipid, membrane thickness and S_CD_ order parameter. Area per lipid and membrane thickness are two important parameters that provide structural insights into membrane properties and contain information about the phase, fluidity, and degree of condensation. In this context, we found that the area per lipid in the presence of the positive modulator is the highest in all simulations, mostly due to alterations in values for DOPE and POPG, which is not likely to be an artifact resulting from the algorithm. Comparing all lipid moieties, simulations with **R1** show a relatively larger area per lipid compared to simulations with **S1**, **R2**, **S2** and simulations without allosteric modulators. This result shows more perturbation in the lipid bilayer in the presence of the positive modulator, which is responsible for more fluctuations in the lipid acyl chains. Our previous results report a key role of the G protein subtype in the activation of the dopamine D_2_ receptor [33]. Some studies indicate that the neutral lipid DOPC can induce partial deactivation of the β_2_-adrenergic receptor [34,35]. The behaviour of this lipid in our simulations may be related to our previous research which showed the dopamine D_2LONG_ receptor in a complex with the G_i1_ protein is partially deactivated. The calculated lateral diffusion coefficient for DOPC also showed a relationship between its value and the type of G protein. The calculations show that DOPC molecules diffuse more easily in the case of systems with the G_i2_ protein. We also noticed the dependence of the coefficient value for cholesterol on the type of the modulator. The lowest value was obtained for the receptor in a complex with the negative allosteric modulator **S1**. Additionally, we investigated the accumulation of cholesterol molecules around the receptor. Our research shows that, depending on the type of modulator, cholesterol molecules are arranged in appropriate clusters and their number is related to the modulation of the receptor. The smallest number of clusters was observed for systems with **S1** and the highest for systems with **R1**.

The next step to study the membrane system was to calculate bilayer thickness. This parameter depends on lipid tilt, carbon chain lengths and the degree of unsaturation of the membrane lipid [36,37]. In our simulations, a significant difference in membrane thickness was observed in the systems with **R1** and **S1** modulators. The values for R1G1 and R1G2 were found to be the lowest among all simulations, while simulations S1G1 and S1G2 show the highest values. The decrease in membrane thickness is accompanied by an increase in the total APL, indicating a decrease in the ordered raft-like character of the bilayer. This may result from alterations in the availability of cholesterol binding sites, hydrophobic mismatch between membrane and hydrophobic regions of TMs, headgroup interactions and mutual lateral pressure, all of these affecting lipid packing in the immediate neighbourhood of the receptor [38]. 

We also measured the orientation and mobility of the C-H bond by calculating the lipid order parameter S_CD_. In a highly ordered state, the acyl chains are set at right angles to the bilayer and have an extended configuration of all atoms with S_CD_ = 0.5. During the simulation, the value can become S_CD_ = 0 in a completely unordered state [39]. Our simulations show differences in the behaviour of individual lipids depending on the combination of the modulator and the type of G_i_ subunit.

The collective interactions between the dopamine D_2_ receptor and G protein heterotrimer with the lipid headgroups in our MD simulations highlight the important role of protein modulation on the lipid membrane and receptor–G protein coupling. We observed some unique interactions of the G protein and 7TM domain of the dopamine D_2_ receptor with lipid headgroups in simulations with positive modulator **R1**. Notably, it was found that residues K10, K17 and R24 from the G protein interact with the polar membrane headgroups of the lipid bilayer in all simulated systems which is in accordance with the experimental results obtained by Yin et al. [32]. For the receptor, the largest changes upon activation in simulation with the **R1** occurred at TM6, which showed movement of T6.34 and E6.30 (residue forming ionic lock), caused by bending and rotation of this helix. This results in exposure of these residues to lipid headgroups, allowing the formation of specific interactions. 

In conclusion, in this article, we discussed how membrane protein modulation affects the surrounding lipids, which may indicate mutual receptor–membrane influence in the context of signalling.

## 4. Materials and Methods

The systems of D_2LONG_ receptor (with ICL3) in complex with respective G protein were built using Modeller v. 9.19 [40] and Yasara Structure v. 20.12.24 [41] tool for loop modelling as previously reported [18,33]. The systems were embedded into a heterogeneous bilayer systems, prepared using the CHARMM-GUI Membrane Builder server [42] in proportions appropriate for membrane rafts [43] and consisting of 31% cholesterol, 18% sphingomyelin, 16% 1-palmitoyl-2-linoleoyl-sn-glycero-3-phosphocholine (PLPC), 12% 1-palmitoyl-2-oleoyl-sn-glycero-3-phosphoethanolamine (POPE), 10% 1-palmitoyl-2-oleoyl-sn-glycero-3-phosphocholine (POPC), 4% 1-Palmitoyl-2-oleoyl-sn-glycero-3-phosphoglycerol (POPG), 4% 1,2-dioleoyl-sn-glycero-3-phosphoethanolamine (DOPE), 4% 1,2-dioleoyl-sn-glycero-3-phospho-L-serine (DOPS) and 1% 1,2-dioleoyl-sn-glycero-3-phosphocholine (DOPC). Hydrated systems (TIP3 water molecules) were neutralized with 0.15 M NaCl. Dopamine was docked with Molegro Virtual Docker 6.0 software [44] and the systems were subjected to 1 μs all-atom MD simulations under periodic boundary conditions using Gromacs v. 2018.4 [45]. The models of D_2L_ receptor in complex with G_i1_ protein and D_2L_ receptor in complex with G_i2_ protein were used in docking simulations to identify allosteric binding sites of modulators. Modulator structures were modelled using the Hartree–Fock approach and 6-31G* basis set of Spartan v. 10 VI.0.1 [46]. The probable allosteric binding pockets were found by docking performed by Molegro Virtual Docker 6.0 software [44]. The poses with the lowest value of the scoring function (MolDockScore) were further analysed. Five potential allosteric pockets were selected for each ligand and several best results of docking in each of these pockets were simulated for 200 ns with Gromacs. An Amber03 force field [47] was used for protein, Slipids (Stockholm lipids) [48] for the membrane and General Amber Force Field (GAFF) [49] for ligands. The most energetically favourable poses were subjected to 1 μs all-atom molecular dynamics in triplicate. As a reference, systems without a modulator (with dopamine) were also simulated. This detailed study involved homology modelling of the D_2L_ receptor in complex with the respective G protein, docking allosteric ligands and 1 μs all-atom MD simulations of the systems used in this study, as described in our previous works [18,33]. Due to the repeatability of the results among the replicas of a given model, this work summarizes the behaviour of the lipid membrane for one of the replicas of a given system. VMD v. 1.9.3 [50], PyMol v. 4.6 [51] and Schrödinger Maestro v. 12.4 software [52] were used for data extraction and analysis of the results. For the analysis of lipid bilayer (surface area per lipid, bilayer thickness and deuterium order parameters) default settings of MEMBPLUGIN 1.1 [53] were used. 

## Figures and Tables

**Table 1:** Values of average area per lipid estimation for systems in the absence (DG1, DG2) and presence of modulators **R1**, **S1**, **R2** and **S2** with the respective G proteins.

Area per Lipid (Å^2^)										
	CHL	SM	DOPE	DOPC	DOPS	PLPC	POPC	POPE	POPG	Total
R1G1	31.02 ± 0.94	42.06 ± 1.03	62.10 ± 1.34	62.32 ± 0.98	58.40 ± 1.05	67.17 ± 1.10	61.18 ± 1.14	54.23 ± 1.32	63.15 ± 1.19	58.46 ± 0.20
R1G2	31.50 ± 0.82	42.59 ± 1.09	62.82 ± 1.09	62.42 ± 0.98	54.01 ± 0.98	66.19 ± 1.11	59.33 ± 1.05	55.46 ± 1.32	56.16 ± 1.23	56.89 ± 0.27
S1G1	30.91 ± 0.87	42.65 ± 1.05	53.57 ± 1.11	57.60 ± 0.99	43.47 ± 1.07	63.90 ± 0.94	52.14 ± 1.02	50.41 ± 1.15	53.60 ± 1.25	48.55 ± 0.33
S1G2	30.60 ± 1.00	43.44 ± 1.12	52.81 ± 1.09	59.14 ± 1.02	51.57 ± 0.92	63.86 ± 1.06	55.23 ± 1.05	50.95 ± 1.19	54.62 ± 1.21	48.40 ± 0.20
R2G1	30.97 ± 1.90	42.42 ± 1.15	57.69 ± 1.43	56.57 ± 1.13	54.12 ± 0.14	63.97 ± 1.26	60.95 ± 1.22	53.24 ± 1.13	54.35 ± 1.40	50.89 ± 0.21
R2G2	31.11 ± 0.82	43.68 ± 1.12	52.65 ± 1.09	57.78 ± 0.93	53.57 ± 1.32	62.97 ± 1.01	55.23 ± 0.98	55.51 ± 0.94	54.35 ± 1.24	50.13 ± 0.32
S2G1	30.89 ± 1.13	42.98 ± 1.09	57.60 ± 1.20	56.97 ± 0.87	52.41 ± 1.07	63.81 ± 1.21	60.83 ± 1.09	53.98 ± 1.23	53.38 ± 1.19	50.47 ± 0.12
S2G2	30.23 ± 1.15	43.65 ± 1.09	53.14 ± 1.02	57.24 ± 0.92	53.20 ± 1.29	62.98 ± 1.10	54.99 ± 0.84	54.39 ± 1.36	55.69 ± 1.12	50.33 ± 0.12
DG1	31.96 ± 1.02	43.06 ± 1.11	57.78 ± 1.11	57.67 ± 0.99	52.36 ± 1.04	64.01 ± 1.05	61.82 ± 1.11	54.54 ± 1.20	53.88 ± 1.23	50.00 ± 0.14
DG2	30.07 ± 1.01	43.26 ± 1.02	53.70 ± 1.21	58.36 ± 0.87	53.36 ± 1.01	63.07 ± 1.04	55.62 ± 0.94	54.58 ± 1.21	55.58 ± 1.31	50.00 ± 0.19

**Table 2:** Values of standard deviation of DOPC APL in different model systems.

System	R1G1	R1G2	S1G1	S1G2	R2G1	R2G2	S2G1	S2G2	DG1	DG2
St. dev.	7.4	4.3	7.3	4.0	7.4	3.9	6.5	4.1	4.6	3.8

**Table 3:** Values of the lateral diffusion coefficient for cholesterol and DOPC molecules in different model systems.

D_2D_ [m^2^/s] × 10^−11^										
System	R1G1	R1G2	S1G1	S1G2	R2G1	R2G2	S2G1	S2G2	DG1	DG2
Cholesterol	3.0	3.4	2.6	2.4	3.0	3.5	3.1	3.5	3.7	3.3
DOPC	1.2	2.0	0.8	3.8	2.1	2.7	1.7	3.6	3.0	3.6

**Table 4:** The number of residues in close contacts with cholesterol.

System	R1G1	R1G2	S1G1	S1G2	R2G1	R2G2	S2G1	S2G2	DG1	DG2
Average minimum distance	0.18	0.17	0.19	0.20	0.18	0.19	0.18	0.19	0.19	0.19
Number of residues in close contact	169	176	149	151	155	158	155	160	123	134

## Data Availability

Not applicable.

## Supplementary Materials

The following are available online, Figure S1: Area per lipid estimated for the R1G1, R1G2, S1G1, S1G2, DG1 and DG2 systems, Figure S2: Area per lipid estimated for the R2G1, R2G2, S2G1 and S2G2 systems, Figure S3: Mean-square displacement (MSD) curves of cholesterol and DOPC lipids for the R1G1, R1G2, S1G1, S1G2, R2G1, R2G2, S2G1, S2G2, DG1 and DG2 systems, Figure S4: The time evolution of the number of contacts of cholesterol and protein within a given distance 0.6 nm in the R1G1, R1G2, S1G1, S1G2, R2G1, R2G2, S2G1, S2G2, DG1 and DG2 systems.

## Abbreviations

DG1	D_2LONG_ receptor in complex with G_i1_ protein
DG2	D_2LONG_ receptor in complex with G_i2_ protein
DOPC	1,2-dioleoyl-sn-glycero-3-phosphocholine
DOPE	1,2-dioleoyl-sn-glycero-3-phosphoethanolamine
DOPS	1,2-dioleoyl-sn-glycero-3-phospho-L-serine
ECL	Extracellular loop
GPCRs	G protein coupled receptors
ICL	Intracellular loop
MD	Molecular dynamics
NAM	Negative allosteric modulator
PAM	Positive allosteric modulator
POPC	1-palmitoyl-2-oleoyl-sn-glycero-3-phosphocholine
POPE	1-palmitoyl-2-oleoyl-sn-glycero-3-phosphoethanolamine
POPG	1-palmitoyl-2-oleoyl-sn-glycero-3-phosphoglycerol
PLPC	1-palmitoyl-2-linoleoyl-sn-glycero-3-phosphocholine
R1	(1,3-benzothiazol-2-yl(2-methyl-2,3-dihydro-indol-1-yl)methanone; enantiomer R
R1G1	compound **R1** bound to D_2LONG_ dopamine receptor in complex with G_i1_ protein
R1G2	compound **R1** bound to D_2LONG_ dopamine receptor in complex with G_i2_ protein
R2	(4-methoxy-1-methyl-*1H*-indol-2-yl)(2-methyl-2,3-dihydro-*1H*-indol-1-yl)methanone; enantiomer R
R2G1	compound **R2** bound to D_2LONG_ dopamine receptor in complex with G_i1_ protein
R2G2	compound **R2** bound to D_2LONG_ dopamine receptor in complex with G_i2_ protein
S1	(1,3-benzothiazol-2-yl(2-methyl-2,3-dihydro-indol-1-yl)methanone; enantiomer S
S1G1	compound **S1** bound to D_2LONG_ dopamine receptor in complex with G_i1_ protein
S1G2	compound **S1** bound to D_2LONG_ dopamine receptor in complex with G_i2_ protein
S2	(4-methoxy-1-methyl-*1H*-indol-2-yl)(2-methyl-2,3-dihydro-*1H*-indol-1-yl)methanone; enantiomer S
S2G1	compound **S2** bound to D_2LONG_ dopamine receptor in complex with G_i1_ protein
S2G2	compound **S2** bound to D_2LONG_ dopamine receptor in complex with G_i2_ protein
