# Supplementary material for: The Role of Lipids in Allosteric Modulation of Dopamine D2 Receptor—In Silico Study
Source: Molecules. 2022 Feb 16;27(4):1335. doi: 10.3390/molecules27041335 (PMC8874991; doi:10.3390/molecules27041335)
Supplement: Supplementary file 1 [file molecules-27-01335-s001.zip › molecules-1555420-SI.pdf]

Supporting Information

The Role of Lipids in Allosteric Modulation of Dopamine D_2 Receptor—In Silico Study

Justyna Żuk [1], Damian Bartuzi [1], Przemysław Miszta [2] and Agnieszka A. Kaczor [1,3,*]

[1] Department of Synthesis and Chemical Technology of Pharmaceutical Substances with Computer Modelling Laboratory, Faculty of Pharmacy, Medical University of Lublin, 4A Chodźki St., PL-20093 Lublin, Poland; j.siudem@gmail.com (J.Ż.); damian.bartuzi@umlub.pl (D.B.)

[2] Faculty of Chemistry, Biological and Chemical Research Centre, University of Warsaw, PL-02093 Warsaw, Poland; pmfenix@wp.pl

[3] School of Pharmacy, University of Eastern Finland, Yliopistonranta 1, P.O. Box 1627, FI-70211 Kuopio, Finland

* Correspondence: agnieszka.kaczor@umlub.pl; Tel.: +48-81-448-72-73

Figures S1–S4

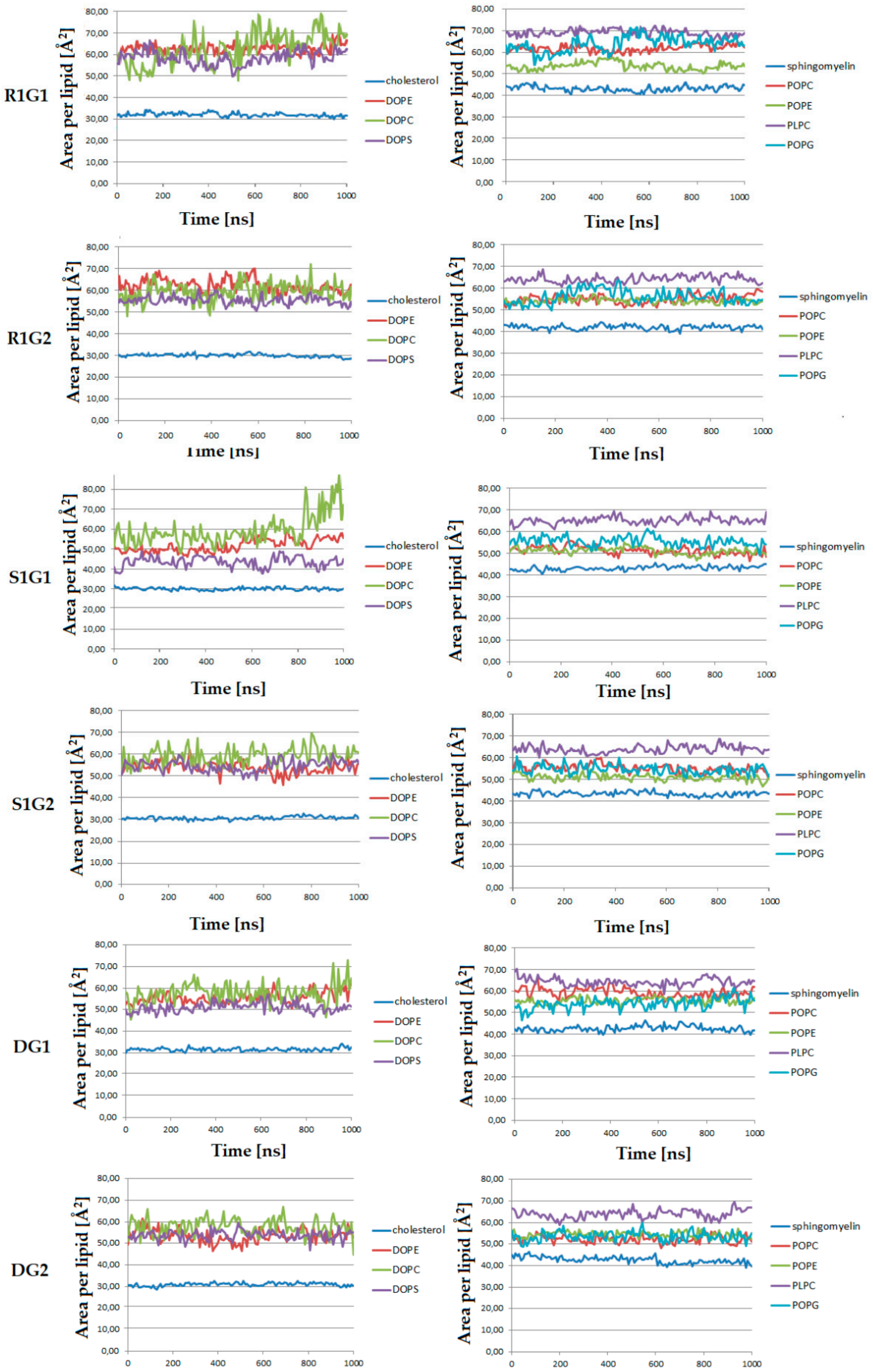

Figure S1. Area per lipid estimated for the R1G1, R1G2, S1G1, S1G2, DG1 and DG2 systems.

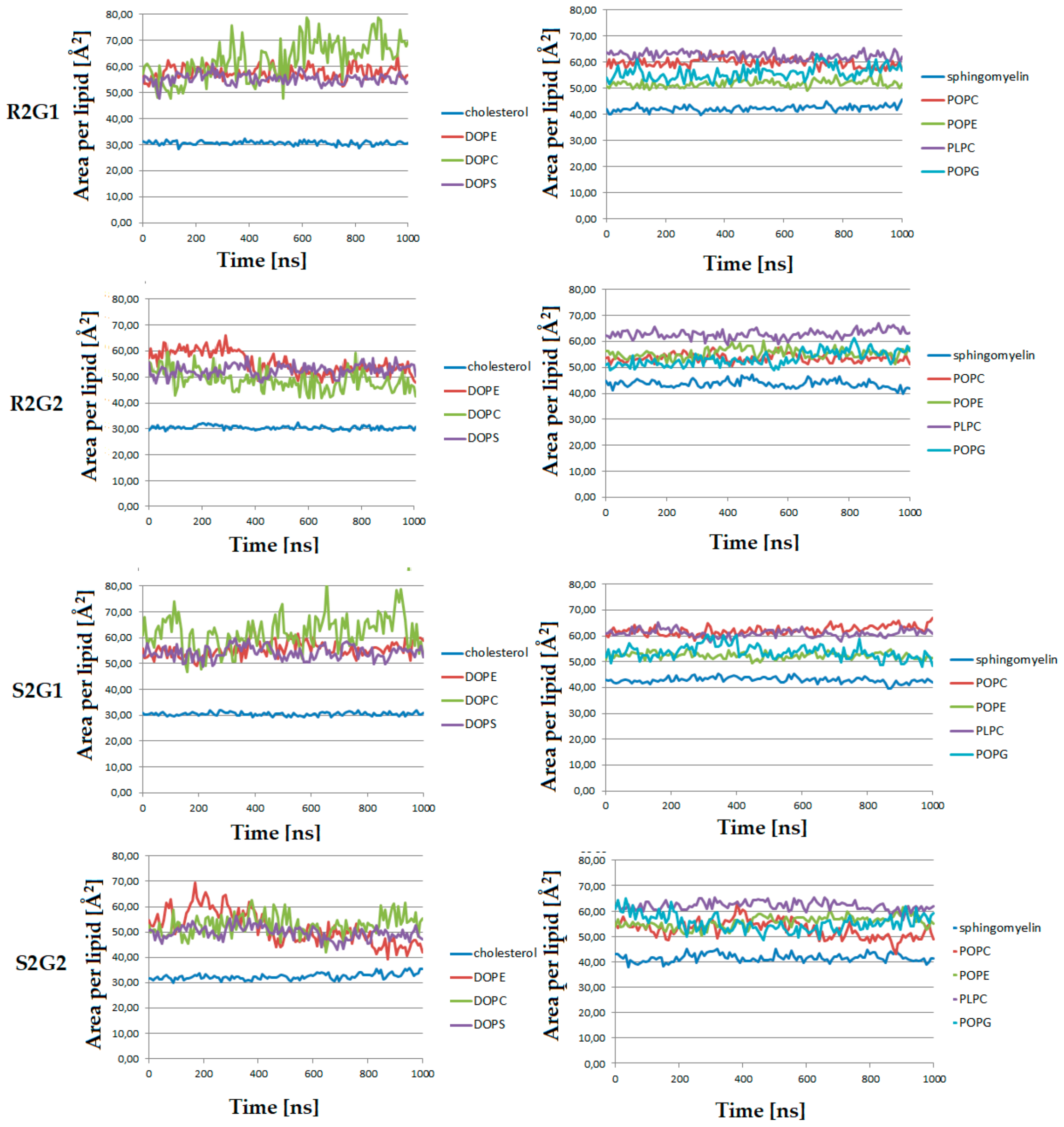

Figure S2. Area per lipid estimated for the R2G1, R2G2, S2G1 and S2G2 systems.

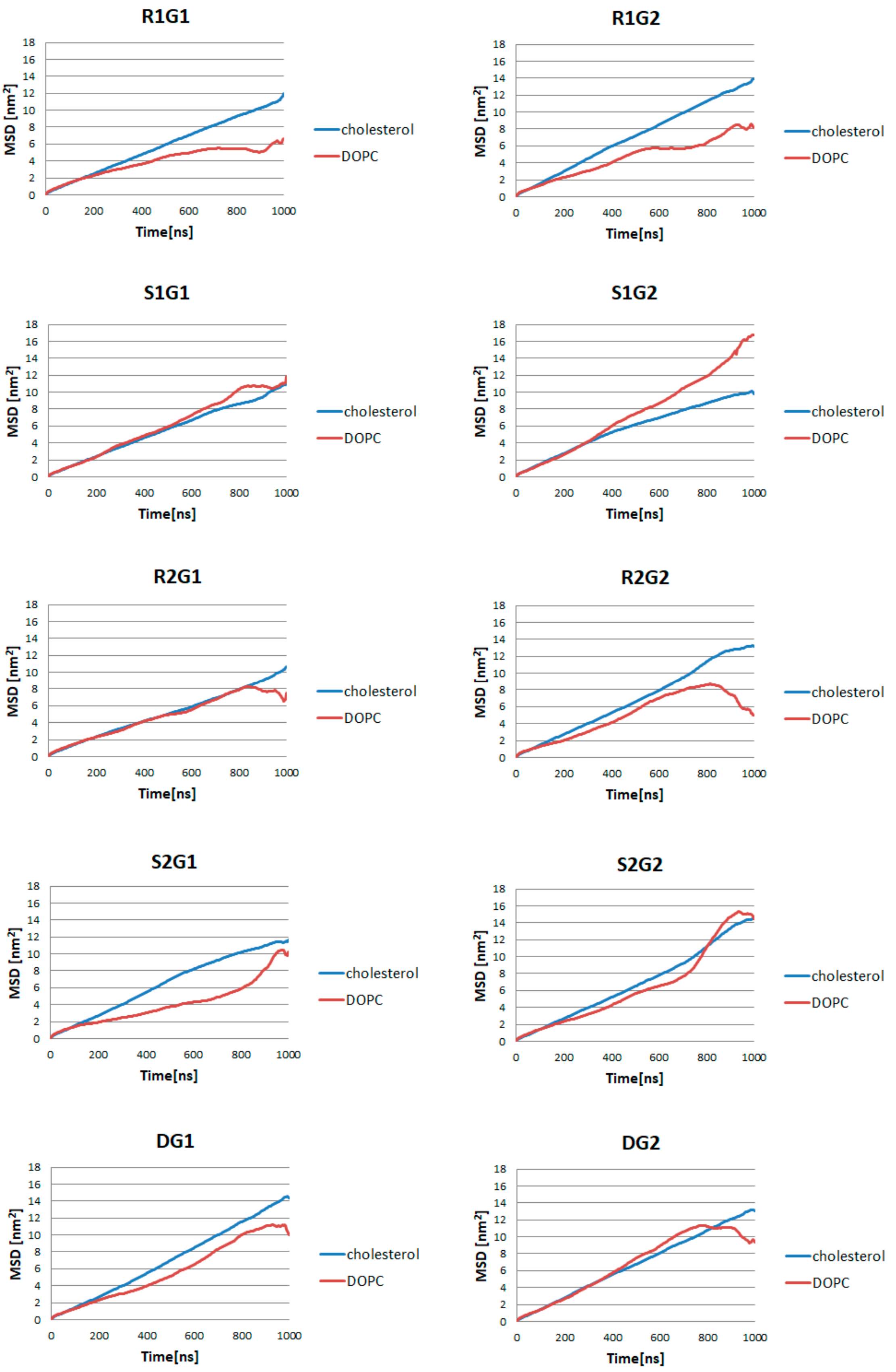

Figure S3. Mean-square displacement (MSD) curves of cholesterol and DOPC lipids for the R1G1, R1G2, S1G1, S1G2, R2G1, R2G2, S2G1, S2G2, DG1 and DG2 systems.

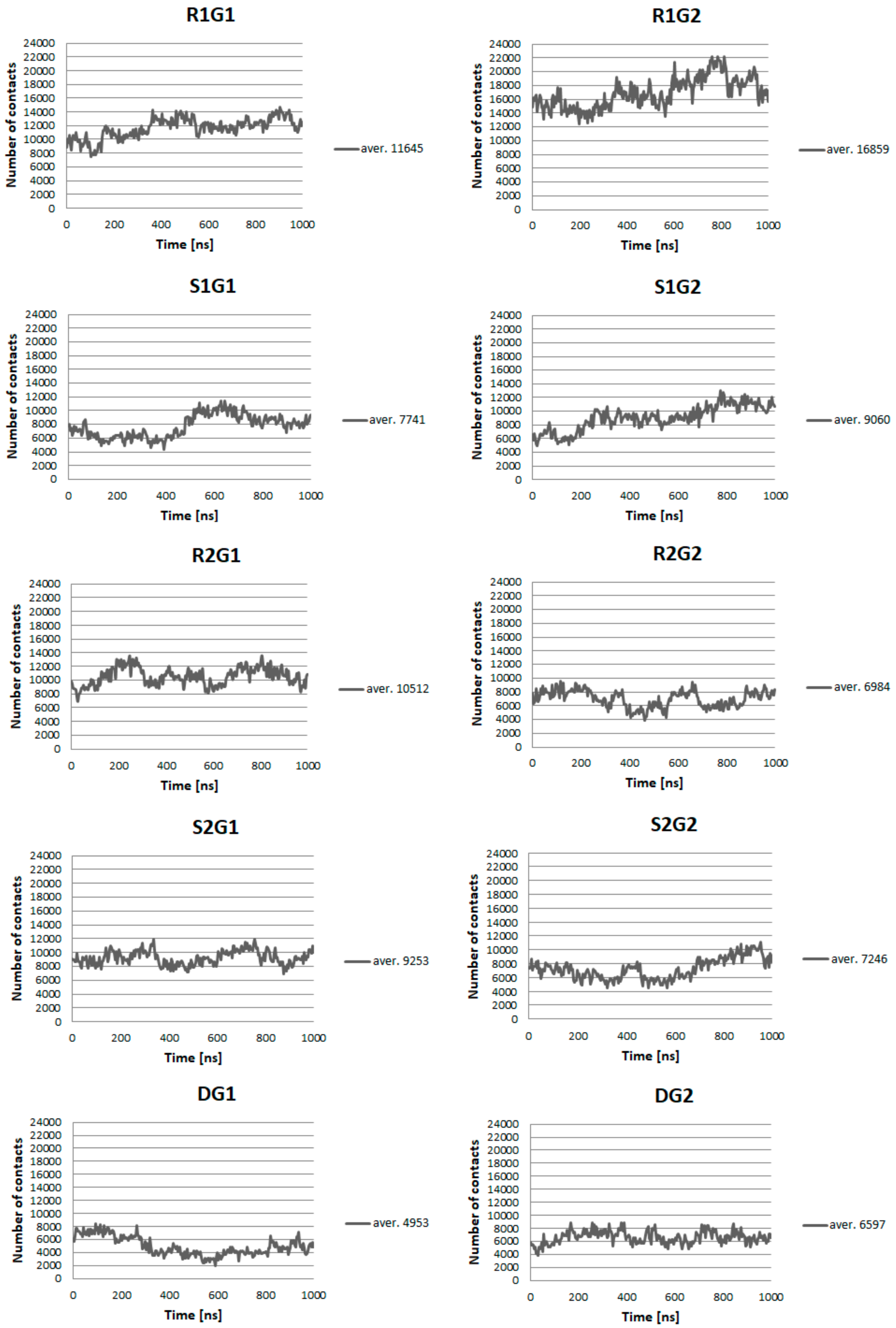

Figure S4. The time evolution of the number of contacts of cholesterol and protein within a given distance 0.6 nm in the R1G1, R1G2, S1G1, S1G2, R2G1, R2G2, S2G1, S2G2, DG1 and DG2 systems.
